# Dyptheritic Cystitis

**Published:** 1882-09

**Authors:** 


### DYPHTHERITIC CYSTITIS.

A very curious case of diphtheritic cystitis is reported by Dr. Jacobi (*N. Y. Medical Journal*, September). The patient has had urinary trouble for a long time ; his urine was frequently very offensive, containing blood and puss. About five days before his death he suddenly collapsed. The doctor found the bladder well filled, and introduced a catheter, but succeeded in removing but a few drops of a gollinsh fetid liquid. Assuming the presence of a malignant tumor at the neck of the bladder, he attempted to draw off the urine by puncturing above the symphisis pubis—again without success. At the post mortem examination a thick membranous lining of the bladder was found detached, in the form of a sac, containing about a quart of urine. During life the beak of the catheter evidently passed into the space between the bladder and the membranous sac, which accounts for the unsuccessful attempts at catheterisation.

